# Efficiency of Plasticity Correction in the Hole-Drilling Residual Stress Measurement

**DOI:** 10.3390/ma13153396

**Published:** 2020-07-31

**Authors:** Tomáš Návrat, Dávid Halabuk, Petr Vosynek

**Affiliations:** Institute of Solid Mechanics, Mechatronics and Biomechanics, Faculty of Mechanical Engineering, Brno University of Technology, Technická 2896/2, 61669 Brno, Czech Republic; navrat@fme.vutbr.cz (T.N.); vosynek@fme.vutbr.cz (P.V.)

**Keywords:** residual stress, hole-drilling method, plasticity effect, finite element method, computational simulation

## Abstract

This paper focuses on the analysis of the plasticity effect in the measurement of the residual stress by the hole-drilling method. Relaxed strains were evaluated by the computational simulation of the hole-drilling experiment using the finite element method. Errors induced by the yielding were estimated for uniaxial tension, plane shear stress state and equi-biaxial stress state at various magnitudes of residual stress uniformly distributed along the depth. The correction of the plasticity effect in the evaluation of residual stress was realized according to the method proposed by authors from the University in Pisa, which was coded in MATLAB. Results obtained from the MATLAB script were compared to the original input data of the hole-drilling simulation and discussed. The analyses suggested that the plasticity effect is negligible at the ratio of applied equivalent stress to yield stress, being 0.6, and that the correction of the plasticity effect is very successful at the previous ratio, being 0.9. Failing to comply with the condition of the strain gauge rosette orientation according to the principal stresses directions causes an increase in the relative error of corrected stresses only for the case of uniaxial tension. It affects the relative error negligibly for the plane shear and equi-biaxial stress states.

## 1. Introduction

Almost all technological operations induce residual stress. These might also occur during the operation of the structure. Its existence negatively influences the generation of various limit states regarding the component failure as well as undesirable changes in the structure shape. Therefore, there is the demand for finding the magnitude of residual stress and adopting measures for their minimization.

**Figure 1:**
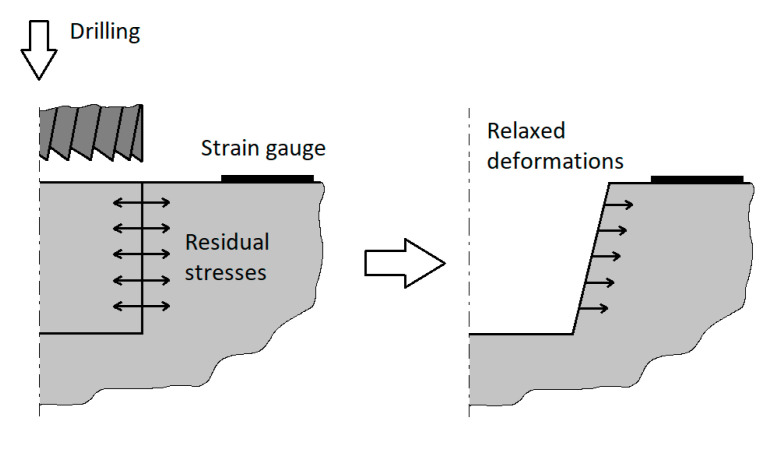
The principle of the hole-drilling method [2].

One of the most frequent methods for measuring residual stress is the semi-destructive hole-drilling method. The drilling of a hole (blind or through) causes the redistribution of residual stress around it (Figure 1). Then, the relaxed strains are measured on the component’s surface by a strain gauge rosette, usually consisting of three resistive grids. The magnitudes of principal residual stresses and respective principal stresses directions can be then evaluated using calibration constants determined by the Finite Element Method (FEM). The necessary condition is the presence of the elastic state of the stress in the investigated section [1].

Plastic deformations develop at the drilled surface and adjacent volume due to stress concentration if the residual stress reaches a certain level. This causes an overestimation of present residual stress and it leads to a certain error of estimated results. The material model and the character of the stress state quantified by, for instance, a biaxiality ratio, also play a role.

Many publications paid attention to the estimation of errors due to the plastic strains around the hole either on the basis of experiments or computational simulations. Beaney and Procter [3] experimentally estimated that the error is negligible for residual stresses under 50% of the yield stress using the four-point bending test. Gibmeier et al. [4] presented the error of 35% for stress equal to 95% of the yield stress, the error of 27% at 80% of the yield stress and the error of 13% at 70% of the yield stress. Therefore, the error started to increase for stresses exceeding 60%–70% of the yield stress at an equi-biaxial stress state. Lin and Chou [5] stated that the error induced by the local plasticity is negligible for residual stresses lower than 65% of the yield stress. The maximum error of 32%–47% occurred for tensile stresses on the level of 95% of the yield stress. Maximum error was reached for elastic–plastic material without hardening (practically with very low tangent modulus). The error values were plotted in dependence on the level of residual stress for low carbon steel, stainless steel and aluminum alloy. Nickola [6] found that the error was negligible for residual stresses lower than 70% of the proportional limit and through hole, while the error was 20%–30% for stresses equal to the yield stress. Vangi and Ermini [7] proved that the simple correction of calibration coefficients does not include the influence of biaxiality as well as the angle between the principal direction and one measuring the grid of the strain gauge rosette. Weng and Lo [8] based on their own experiments the conclusion that the calibration coefficients are almost constant (the plasticity effect is very small) for residual stresses up to 70% of the yield stress. Kornmeier et al. [9] presented that the integral method overestimates the residual stresses by 10%–20% for residual stresses exceeding 95% of the yield stress.

It can be concluded that all the above results obtained at various points and with difficult comparable conditions do not provide a reliable answer to the question; which magnitudes of residual stress produce still acceptable errors in the engineering perspective? Generally, it is assumed that the results of residual stress measurements are reliable when the equivalent residual stress did not exceed 60% of the yield stress. The limits given by the ASTM standard [10] are considered reliable: 50% of the yield stress for thin-walled structures and 80% of the yield stress for thick-walled structures. It is 60% of the yield stress regardless of the structure thickness within the 2008 version of the ASTM standard.

Possible corrections of the plasticity effect in evaluating the residual stress can be found in numerous works. Yan et al. [11] proposed a critical parameter of the plastic deformations at the hole edge under the assumption of the elastic stress state. A simple correction function was presented based on this parameter in order to correct the effect of plasticity. Wang and Huang [12] divided the residual stresses in to four intervals (when the plastic deformations develop around the hole) and experimentally estimated the respective calibration coefficients for them using the pulled specimens. Moharami [13] conducted extensive simulations by means of FEM (more than one thousand analyses) covering eleven variants of the tangent and elastic moduli ratio, ten variants of the maximum to yield stresses ratio and nine variants of the maximum to minimum stresses ratio. Obtained results were approximated by a simple formula for correcting the evaluated principal residual stresses according to the ASTM method. Vangi and Tellini [14] used a computation model of elastic–plastic material in a combination with iterative FEM computations, which gives the possibility of considering various mechanical characteristics (elastic modulus, Poisson’s ratio or stress–strain relationship) or dimensions of a strain gauge rosette or hole. Fourier’s series with five terms were used for the description of relaxed strains. It was also shown that better convergence is reached for the strain gauge rosette with four measuring grids. Seifi and Sallimi-Majd [15] introduced two other constants, C and D, for plasticity effect correction, which extended two usual calibration coefficients A and B. Their magnitude was evaluated on the basis of numerical simulations of the wall with a drilled through hole from material with bilinear hardening. It was shown that von Mises equivalent stress overestimates the plasticity effect for residual stress of distinct signs, and, therefore, the equivalent stress was expressed on the basis of weighted averages of the stress intensities.

The aim of this paper is focused on the method developed at the University of Pisa by Beghini et al. [16,17,18]. This method was also implemented within the EVAL 7 software, supplied with the RESTAN-MTS3000 system developed by SINT Technology (version 7.13, Calenzano, Italy). According to the authors, it should be effective for residual stress reaching up to the yield stress. The necessary assumptions of this correction are: two respectively perpendicular resistive grids of the strain gauge rosette have to be oriented along the principal stress directions and the stress state along the depth has to be homogeneous. It is not always possible to orient the strain gauge rosette along the principal stress directions, as these directions may be unknown, or inaccuracies may occur during application of the strain gauge rosette. In addition, the relevant material characteristics may be available for each investigated component. For these reasons, it is important to know how the orientation of the strain gauge rosette or unknown material characteristics affect the relative error of the measured residual stresses.

The main goals of this work can be summarized as follows. To evaluate the errors of residual stress estimation at:

- various magnitudes of residual stress (related to the yield stress),
- various biaxiality ratios of applied stresses (uniaxial tension, plane shear stress state and equi-biaxial stress state),
- various rosette strain gauge orientations according to the principal directions,
- uncertain knowledge of the material constant (yield stress) of the investigated material,
- using rosette strain gauge type 1-RY61-1.5/120S (HBM),
- using the correction of plasticity effect developed at the University in Pisa with inclusion of all the above stated factors.

The above mentioned allows for the following:

- to obtain a qualified opinion on various recommendations related to the maximum value of reliable evaluated residual stresses,
- to assess the errors due to the plasticity effect with using the above-mentioned ASTM standard,
- to verify the quality of the plasticity effect correction method developed by the University in Pisa.

## 2. Materials and Methods

Beghini et al. [16,17,18] published one of the most beneficial works on the hole-drilling method (HDM), where the maximum error stated is of ±4%, typical ones then in the range 1%–2%, for high values of the residual stresses (plasticity factor 0.99) and low strain hardening (the ratio of the tangent modulus $E_{T}$ to elastic modulus $E\;r=E_{T}/E=0.0$, which is the so-called hardening ratio). This method should be effective for cases of residual stresses up to the yield stress according to its authors. The aim is to express the principal residual stresses acting in the elastic–plastic state using the principal residual stresses obtained under the assumption of elastic behavior. The respective equivalent stresses read

(1) $$\sigma_{eq}=f\left(\sigma_{eq,e},\sigma_{eq,i},R_{e},\frac{E_{T}}{E},\Omega ,\frac{z}{D}\right)$$

where $\sigma_{eq,e}$ is elastically evaluated equivalent stress by the ASTM standard, $\sigma_{eq,i}$ is the equivalent residual stress, when the plastic deformation is induced on the cylindrical surface of the drilled hole, $R_{e}$ is yield stress, Ω is the biaxiality ratio (which is the ratio of residual stress in y direction to residual stress in x direction), z is the hole depth and D is the mean diameter of the strain gauge rosette. The equivalent stress can be written as

(2) $$\sigma_{eq}=\sqrt{\sigma_{x}^{2}+\sigma_{y}^{2}-\sigma_{x}\sigma_{y}}=\sigma_{x}\sqrt{1-\Omega +\Omega^{2}}$$

The corrected residual stresses in the plane x and y are, respectively,

(3) $$\sigma_{x}=\frac{\sigma_{eq}}{\sqrt{1-\Omega +\Omega^{2}}}$$

(4) $$\sigma_{y}=\Omega \sigma_{x}$$

Then, the equivalent residual stress (according to von Mises yield criterion) evaluated from the principal residual stresses $\sigma_{x,e}$ and $\sigma_{y,e}$ in the plane x and y under the assumption of elastic material behavior is

(5) $$\sigma_{eq,e}=\sqrt{\sigma_{x,e}^{2}+\sigma_{y,e}^{2}-\sigma_{x,e}\sigma_{y,e}}$$

The biaxiality ratio for elastic stress state is $\left(-1\leq \Omega_{e}\leq 1\right)$

(6) $$\Omega_{e}=\frac{\sigma_{min}}{\sigma_{max}}$$

where $\sigma_{min}$ and $\sigma_{max}$ are chosen from $\sigma_{x,e}$ and $\sigma_{y,e}$ so that the following condition applies

(7) $$\left|\sigma_{min}\right|\leq \left|\sigma_{max}\right|$$

Next, the equivalent residual stress with which the plastic deformation starts to develop on the cylindrical surface of the drilled hole occurs, when for the tangential stress $\left(\sigma_{t}\right)$ on the cylindrical surface applies the following Kirsch’s theory [19]

(8) $$\sigma_{t}=3\sigma_{x,i}-\sigma_{y,i}=\sigma_{x,i}\left(3-\Omega_{e}\right)=R_{e}$$

where $\sigma_{x,i}$ and $\sigma_{y,i}$ are the principal residual stresses in the plane x and y in the moment, when the plastic deformations initiate on the drilled cylindrical hole surface. Then the respective equivalent stress is

(9) $$\sigma_{eq,i}=\sqrt{\sigma_{x,i}^{2}+\sigma_{y,i}^{2}-\sigma_{x,i}\sigma_{y,i}}=R_{e}\frac{\sqrt{1-\Omega_{e}+\Omega_{e}^{2}}}{3-\Omega_{e}}$$

For further analysis, the plasticity factor calculated under the assumption of the elastic behavior is introduced as

(10) $$f_{e}=\frac{\sigma_{eq,e}-\sigma_{eq,i}}{R_{e}-\sigma_{eq,i}}$$

along with the plasticity factor

(11) $$f=\frac{\sigma_{eq}-\sigma_{eq,i}}{R_{e}-\sigma_{eq,i}}$$

which yields in

(12) $$\sigma_{eq}=f\left(R_{e}-\sigma_{eq,i}\right)+\sigma_{eq,i}$$

The correlation relationships between the above plasticity factors were estimated using the extensive numerical simulations by means of FEM, while the equality of the biaxiality parameters was assumed

(13) $$\Omega =\Omega_{e}$$

The following correction expression was considered [18,19]

(14) $$f_{e}=f+Cf^{2}$$

from which the plasticity factor can be directly expressed as

(15) $$f=\frac{\sqrt{1+4Cf_{e}}-1}{2C}$$

It was estimated for the through hole that

(16) $$C=0.793{\left(1-r\right)}^{2}\left(0.6495\sin\left(2\gamma \right)+1\right)$$

while for the blind one the following

(17) C=(0.167−0.281r)(sin(2γ)+0.299−0.390r)

where γ is the following

(18) $$\gamma =\tan^{-1}\left(\Omega \right)$$

The correction in the latter variant was introduced as [18]

(19) $$f_{e}=f+Wf^{\mu }$$

where W and μ are the functions of the normalized hole depth, normalized hole diameter, hardening ratio and biaxiality parameter. The plasticity factor has to be solved iteratively in this case.

The whole algorithm was programmed within MATLAB. It can be summarized into the following bullet points according its sequence:

- $\sigma_{x,e}$ and $\sigma_{y,e}$ are calculated using the relaxed strains under the consideration of linearly elastic state of stress,
- $\sigma_{eq,e}$ is calculated using the Equation (5),
- $\Omega_{e}$ is calculated using the Equation (6),
- $\sigma_{eq,i}$ is calculated using the Equation (9),
- $f_{e}$ is calculated using the Equation (10),
- f is calculated using either the Equation (15) or iterating the Equation (19),
- $\sigma_{eq}$ is calculated using the Equation (12),
- $\sigma_{x}$ is calculated using the Equation (3) considering the Equation (13),
- $\sigma_{y}$ is calculated using the Equation (4) considering the Equation (13).

It can be concluded that there is still unknown practical methodology usable for general cases of the plane stress state with unknown principal directions and with stresses non-uniformly distributed along the hole depth (hence with the stress gradient along the depth).

## 3. Computational Modelling of the Hole Drilling

**Figure 2:**
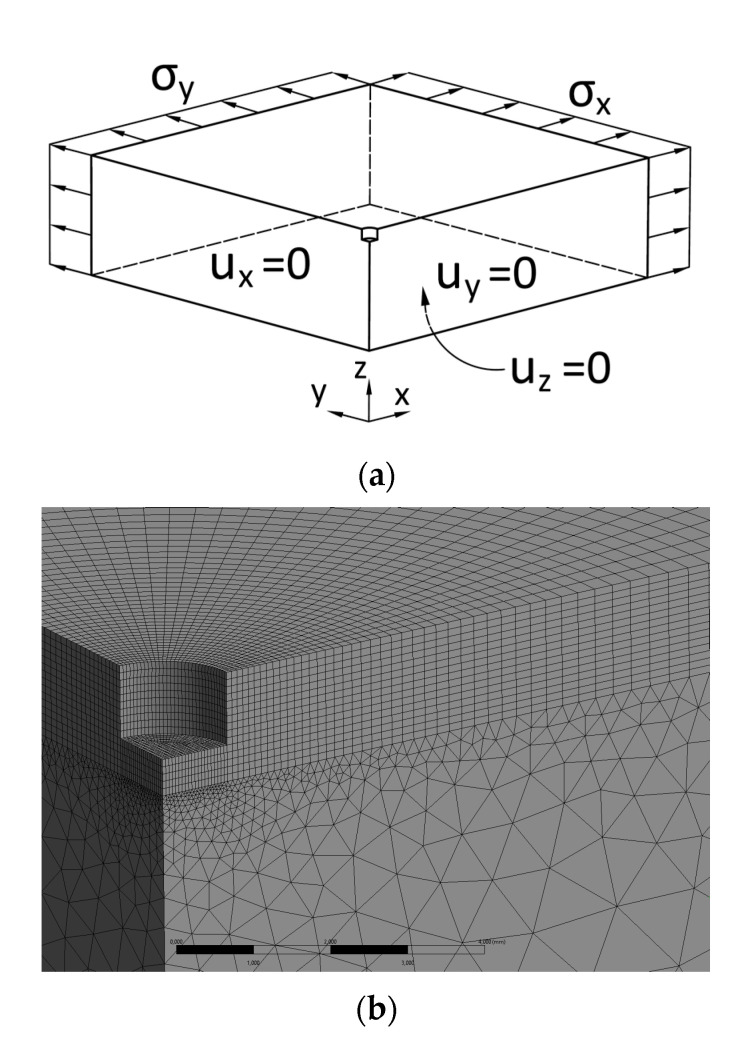
(**a**) Model of geometry with boundary conditions (adapt from [20]); (**b**) the finite element mesh around the drilled hole.

The strains relaxed by the drilling (or milling, respectively) of the hole were obtained using the computational simulations of this process by means of the FEM. The computational model was created in order to investigate the distribution of strains around the drilled hole. The model was based on the procedure “hole after residual stress”, which prescribes the residual stress to the solid, while the relaxed strains are measured after the removal of the material from the hole. The hole-drilling process was simulated by the stepwise deactivating of particular layers of elements. There were used 10 respective layers for the material removal. The nonlinear solution was carried out within the ANSYS software (version 19.0, Canonsburg, PA, USA). The model used in the calculations was a thick solid body with dimensions 60 × 60 × 25 mm. Because of the symmetry, the simulated model consists only of one quarter of the whole geometry. The boundary conditions of zero displacement in the perpendicular direction were applied on the faces of the model in the symmetry planes. Another zero displacement was applied on the bottom face of the model to guarantee the numerical stability of the calculation. The stress in the model, which represents the residual stress, was generated by loading external faces of the model in the x and y direction. The mapped mesh with solid elements SOLID186 was uniformly spaced around the hole and along the depth. Finer mesh (element size 0.05 mm) was used in the area surrounding the hole and coarser mesh (element size 3 mm) was used near the far boundaries. The element size was gradually increased from the hole towards the boundaries. The total number of elements and nodes was 123,000 and 257,000, respectively. Figure 2 depicts the geometry with boundary conditions and the finite element mesh around the drilled hole used in simulations.

**Figure 3:**
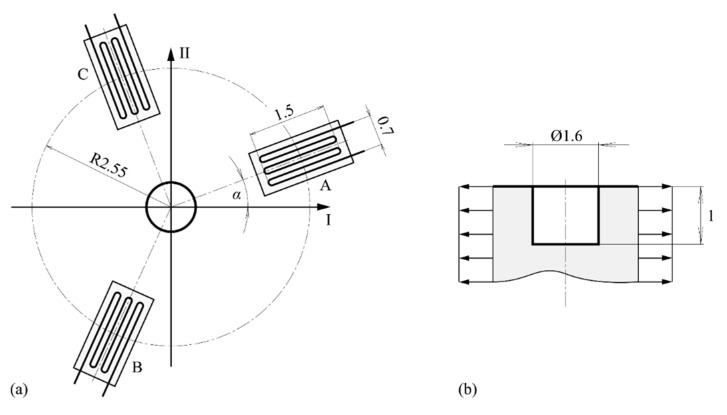
Dimensions in mm of: (**a**) strain gauge rosette type 1-RY61-1.5/120S; (**b**) drilled hole.

The strains were obtained from the virtual strain gauge rosette by averaging of the nodal strains across the strain gauge grid surface at stepwise removal of the hole layers. Dimensions, designations and orientations of the strain gauge rosette according to the directions of applied load are depicted in Figure 3a. The dimensions of the drilled hole are given in Figure 3b.

The bilinear stress–strain relationship and von Mises yield criterion with kinematic hardening and associative flow rule were used for the description of the material behavior. The elastic modulus was 210,000 MPa and Poisson’s ratio was 0.3. The tangent moduli were 2100 MPa, 21,000 MPa and 52,500 MPa, respectively. This helped to quantify the influence of the hardening on the precision of the evaluated residual stress. The yield stress was considered $R_{e}$= 500 MPa.

Computational simulations were realized only for an ideally concentric hole. The elastic–plastic states were induced within the material around the hole for the following states of stress:

- uniaxial tension (biaxiality ratio $\Omega_{e}=0$),
- plane shear stress state (biaxiality ratio $\Omega_{e}=-1$),
- equi-biaxial stress state (biaxiality ratio $\Omega_{e}=1$).

**Figure 4:**
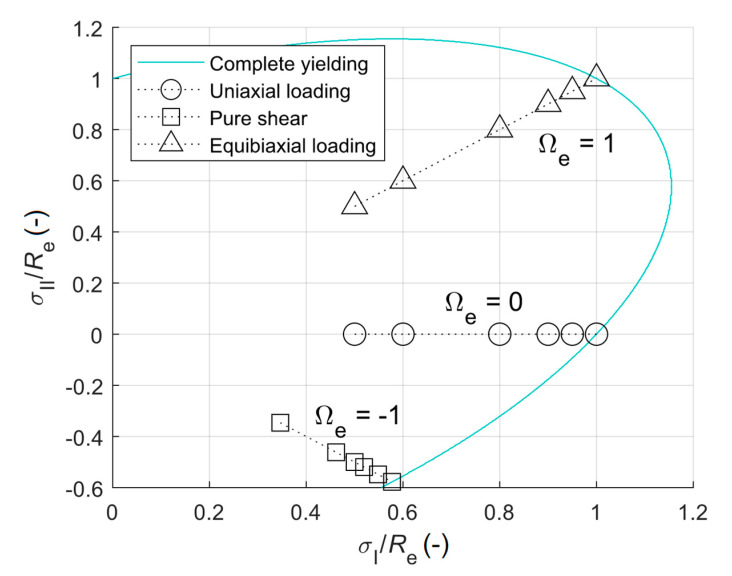
Graphical interpretation of performed computations.

The following was considered:

- ${\bar{\sigma }}_{eq}/R_{e}=0.5$, 0.6, 0.8, 0.9, 0.95 and 1,
- $E_{T}/E=$ 0.01, 0.1, 0.25,
- α=0°, 15°, 30°, 45° and 60°.

where ${\bar{\sigma }}_{eq}$ is the applied equivalent stress according to von Mises yield criterion (stresses in two directions were actually applied in the FEM model). The used combinations of $\sigma_{I}/R_{e}$ with $\sigma_{II}/R_{e}$, where $\sigma_{I}$ and $\sigma_{II}$ are the principal stresses in I and II principal directions, respectively, and biaxiality ratios are shown in Figure 4.

## 4. Results and Discussion

The errors of von Mises equivalent stresses were estimated. The absolute error of the equivalent stresses is

(20) $$∆=\sigma_{eq,e}-{\bar{\sigma }}_{eq}$$

The relative error of evaluated equivalent stress can be expressed as a ratio of an absolute error of this stress and a real (true) value of equivalent stress (the applied one). It is in percent for uncorrected stresses as

(21) $$\delta =100\frac{∆}{{\bar{\sigma }}_{eq}}=100\frac{\sigma_{eq,e}-{\bar{\sigma }}_{eq}}{{\bar{\sigma }}_{eq}}$$

while for the corrected ones as

(22) $$\delta_{cor}=100\frac{\sigma_{eq}-{\bar{\sigma }}_{eq}}{{\bar{\sigma }}_{eq}}$$

Strains computed using FEM were entered into the script in MATLAB and the corrected stresses were evaluated using HDM.

### 4.1. Uniaxial Tension ($\Omega_{e}=0$)

Results obtained for the uniaxial tension and the angle between the grid A of the strain gauge rosette and the applied principal stress α=0° are given in Table 1.

**Figure 5:**
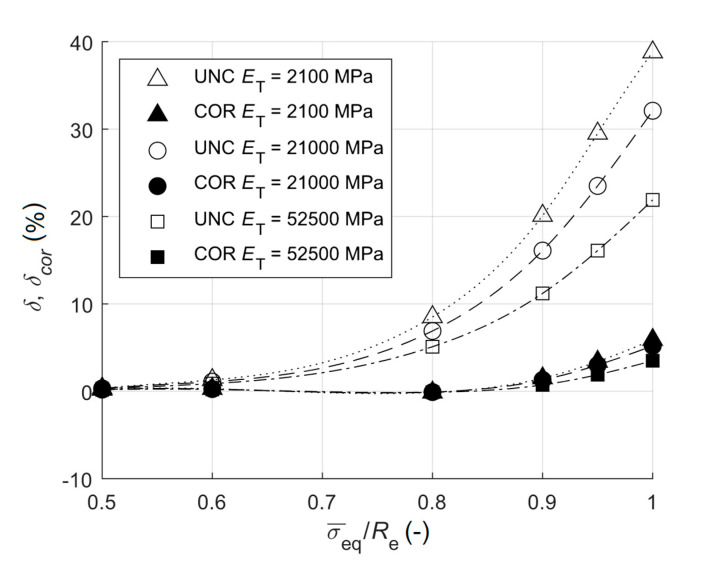
The dependency of the relative error on the ratio of the equivalent stress to yield stress for various strain hardening (UNC states for uncorrected and COR states for corrected).

The error of uncorrected stress increased with the increasing ratio ${\bar{\sigma }}_{eq}/R_{e}$. It is obvious from Table 1 and Figure 5 that the relative error was 5.1%–8.5% for ${\bar{\sigma }}_{eq}/R_{e}=0.8$ depending on the strain hardening (tangent modulus). These errors were significant, therefore the value of 80% of the yield stress should be approached critically. The correction on plasticity within the MATLAB code was still very successful for ${\bar{\sigma }}_{eq}/R_{e}=0.9$, which corresponds to results of Beghini et al. [17]. The relative error, though, slightly increased at higher magnitudes of the residual stress, even after the correction. This error can be considered as acceptable, especially for materials with high strain hardening. The relative errors for materials with higher strain hardening were slightly lower than those for materials with low strain hardening, which was simulated by $E_{T}=2100$ MPa, hence with the ratio $r=E_{T}/E=0.01$.

Then, the errors coming from failing to comply with the demand on the rosette strain gauge orientation along with the principal stress directions were studied. The simulations were done for ratio ${\bar{\sigma }}_{eq}/R_{e}=0.8$ with tangent modulus equal to 2100, 21,000 and 52,500 MPa and for various orientation of the strain gauge rosette. The results are summarized in Table 2.

**Figure 6:**
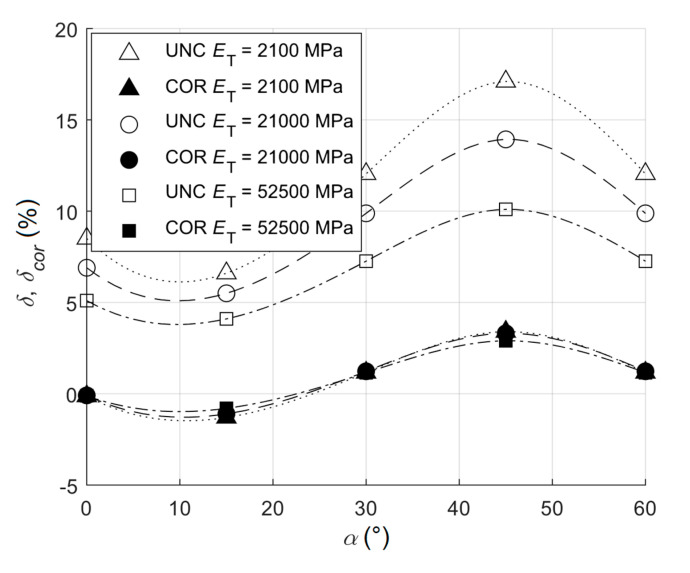
The dependency of the relative error on the angle for the ratio ${\bar{\sigma }}_{eq}/R_{e}=0.8$ (UNC states for uncorrected and COR states for corrected).

The error due to the failure in orienting the strain gauge rosette within the principal directions caused the increase of the relative error for uncorrected stresses as well as corrected ones (Table 2 and Figure 6). This increase was more than double for uncorrected stresses (compared to the corrected ones) and respective errors were high.

Then, the errors coming from the unknown yield stress of the investigated material were also studied. The yield stress is often unknown and has to be approximated. These sources of uncertainties should be accounted for in the analysis of experimental uncertainties. The material was considered having the yield stress $R_{e}=500$ MPa and tangent modulus $E_{T}=2100$ MPa in simulations. Applied residual stress was $\sigma_{I}=400$ MPa, which was equal to 80% of the yield stress. However, these values are not known during the evaluation. The strains obtained by the computational hole-drilling simulations were evaluated under these conditions and used for the evaluation of corrected stresses, assuming the value of the yield stresses $R_{e}^{C}$ being in the interval 450–650 MPa. The obtained results are summarized in Table 3.

The corrected equivalent residual stress was in the interval 383.7–427.9 MPa for used interval of (uncertain) yield stress 450–650 MPa. Range of the residual stress was therefore 44 MPa. The guess was good according to the applied stress of 400 MPa, when the mean value was 406 MPa.

### 4.2. Plane Shear Stress State ($\Omega_{e}=-1$)

**Figure 7:**
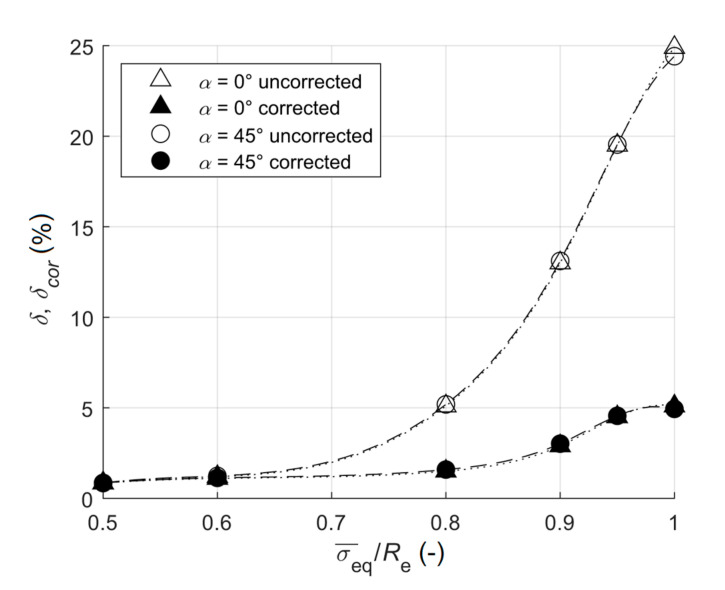
The dependence of the relative error on the ratio of the equivalent stress to yield stress for plane shear stress state.

**Figure 8:**
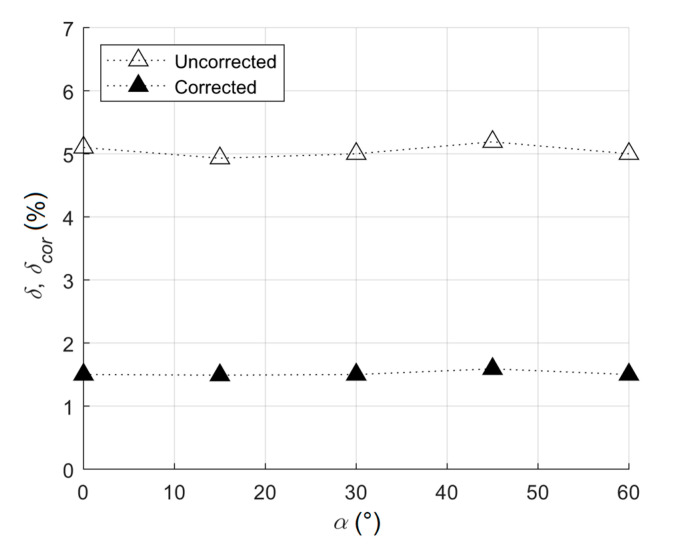
The dependence of the relative error on the angle α for the plane shear stress state angle for the ratio ${\bar{\sigma }}_{eq}/R_{e}=0.8$.

The results are summarized in Table 4 and Figure 7 for ratios ${\bar{\sigma }}_{eq}/R_{e}=0.5$, 0.6, 0.8, 0.9, 0.95 and 1 for plane shear stress state (pure shear), and also for the orientation of the strain gauge rosette α= 0° and 45°, while the results in Table 5 and Figure 8 are for the strain gauge rosette rotated 0, 15, 30, 45 and 60 degrees to the principal stress directions and for ${\bar{\sigma }}_{eq}/R_{e}=0.8$.

The errors of uncorrected equivalent stresses were lower for the plane shear stress state, when compared to uniaxial tension. These differences increased with the increasing magnitude of the residual stress (Figure 7). On the contrary, the errors of corrected equivalent stresses were higher for the plane shear stress state, when compared to uniaxial tension. Therefore, the plasticity correction was less efficient at the plane shear stress state than that at uniaxial tension. However, the errors were still acceptable (Figure 7).

The magnitudes of errors only slightly changed for the rotated strain gauge rosette (Table 5 and Figure 8). Therefore, unknown principal stress directions did not affect the error of the result from the practical point of view for the plane shear stress state.

### 4.3. Equi-Biaxial Stress State ($\Omega_{e}=1$)

Results obtained for the concentric hole and assessment of the influence of plasticity correction is given in Table 6. Material parameters $E_{T}=2100$ MPa and $R_{e}=500$ MPa and the orientation of the strain gauge rosette α= 0° were used in the computations.

**Figure 9:**
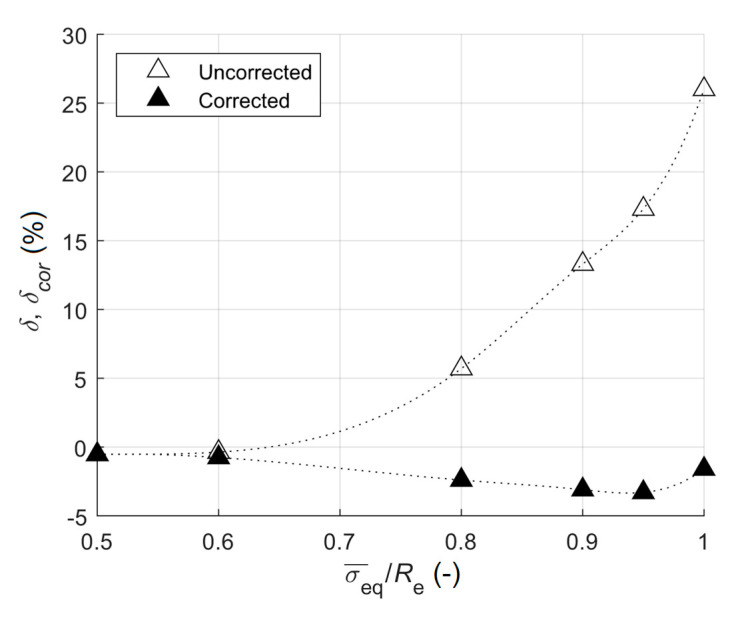
The dependence of the relative error on the ratio of equivalent stress to yield stress for equi-biaxial stress state.

Errors of uncorrected equivalent stresses for the equi-biaxial stress state (equi-biaxial tension) were lower than those for the uniaxial tension, the same situation as in the case of the plane shear stress state. These differences increased as the magnitude of residual stress arose (Figure 9). As all the stress directions were the principal ones in this case, the results were not influenced by the angle α.

### 4.4. The Comparison of All Stress States

**Figure 10:**
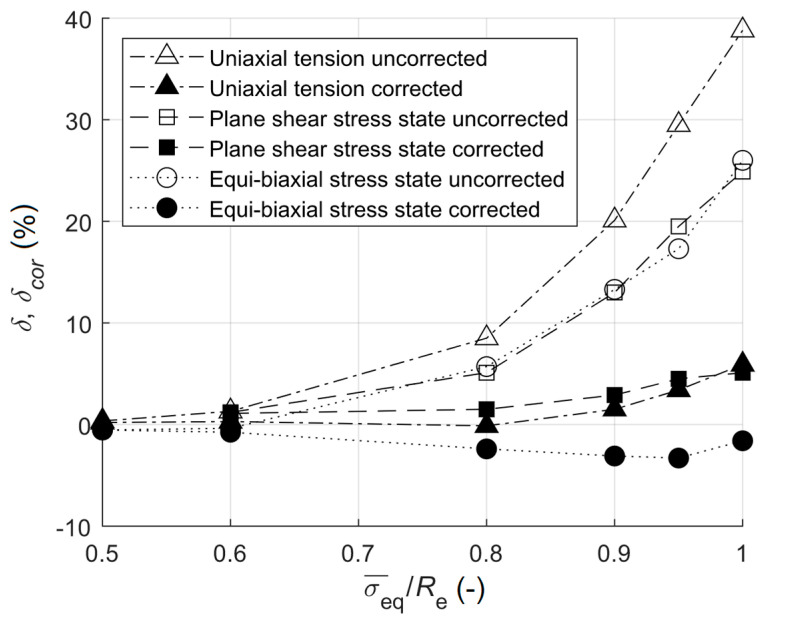
The dependence of the relative error on the ratio of equivalent stress to yield stress for all modelled stress states ($E_{T}=2100$ MPa).

The comparison of errors for all stress states with $E_{T}=2100$ MPa is shown in Figure 10. Errors of uncorrected equivalent stresses are higher for uniaxial tension than for the plane shear or equi-biaxial stress state. On the contrary, errors of corrected equivalent stresses were higher for the equi-biaxial stress state and the plane shear stress state, when compared to uniaxial tension. Therefore, the plasticity correction for the equi-biaxial stress state and the plane shear stress state was less successful than in the case of uniaxial tension, except in the case where ${\bar{\sigma }}_{eq}/R_{e}=1$. Despite that, the errors of corrected equivalent stresses were very low.

## 5. Conclusions

The effect of plasticity was analyzed for measurement of the residual stress using the hole-drilling method. The main goal of the research was to investigate the efficiency of the plasticity correction in the hole-drilling method for industrial practice; that means for states, where correct orientation of the strain gauge rosette is not always guaranteed or the yield strength of material may be unknown. All the simulations assumed the uniformly distributed stress along with depth, while three different stress states were investigated (uniaxial tension, plane shear stress state and equi-biaxial stress state). The main conclusions are summarized as follows:

- The plasticity effect was negligible for residual stress ratio lower than ${\bar{\sigma }}_{eq}/R_{e}=0.6$.
- The correction on the plasticity effect was very successful at ${\bar{\sigma }}_{eq}/R_{e}=0.9$ for the hole-drilling method programmed within MATLAB. The relative error increased for higher magnitudes of residual stress. Nevertheless, the correction can be still considered as acceptable.
- Failing to comply with the demand on the strain gauge rosette orientation along with the principal stress directions caused an increase in the relative error for corrected stress only in the case of uniaxial tension. Nevertheless, the relative errors are still acceptable from an engineering point of view. Unknown principal stress directions influenced only slightly the error for the plane shear and equi-biaxial stress state.
- The unknown yield strength of the material affected the efficiency of the correction method, but if the yield strength used for the correction method was in range of ±100 MPa from its actual value, the relative error for the uniaxial tension stress state was up to 5%.

## Figures and Tables

**Table 1:** Relative errors of chosen evaluated residual stresses for uniaxial tension.

α (°)	$\sigma_{I}$ (MPa)	${\bar{\sigma }}_{\mathrm{eq}}/R_{e}$ (-)	$E_{T}$ (MPa)	δ (%)	$\delta_{cor}$ (%)
0	250	0.5	2100	0.35	0.20
			21,000	0.33	0.19
			52,500	0.29	0.17
	300	0.6	2100	1.3	0.29
			21,000	1.1	0.25
			52,500	0.88	0.21
	400	0.8	2100	8.5	–0.13
			21,000	6.9	–0.09
			52,500	5.1	–0.11
	450	0.9	2100	20.1	1.5
			21,000	16.1	1.3
			52,500	11.2	0.73
	475	0.95	2100	29.5	3.4
			21,000	23.5	3.0
			52,500	16.1	1.9
	500	1	2100	38.8	5.9
			21,000	31.8	5.2
			52,500	21.9	3.5

**Table 2:** Relative errors of chosen evaluated residual stress for uniaxial tension and various angles between the grid A of the strain gauge rosette and the applied principal stress.

α (°)	$\sigma_{I}$ (MPa)	${\bar{\sigma }}_{\mathrm{eq}}/R_{e}$ (-)	$E_{T}$ (MPa)	δ (%)	$\delta_{cor}$ (%)
400	0.8	0	2100	8.5	−0.13
			21,000	6.9	−0.09
			52,500	5.1	−0.11
		15	2100	6.6	−1.3
			21,000	5.5	−1.1
			52,500	4.1	−0.8
		30	2100	12.04	1.16
			21,000	9.88	1.23
			52,500	7.26	1.15
		45	2100	17.1	3.4
			21,000	13.93	3.31
			52,500	10.1	2.9
		60	2100	12.04	1.16
			21,000	9.88	1.23
			52,500	7.26	1.15

**Table 3:** Relative errors of evaluated residual stresses for $R_{e}^{C}$ in the interval 450–650 MPa.

$R_{e}^{C}/R_{e}$ (-)	$R_{e}^{C}$ (MPa)	$E_{T}$ (MPa)	δ (%)	$\sigma_{eq}$ (MPa)	$\delta_{cor}$ (%)
0.9	450	2100	8.5	383.7	–4.1
1	500		8.5	399.5	–0.13
1.1	550		8.5	411.2	2.8
1.2	600		8.5	419.6	4.9
1.3	650		8.5	425.2	6.3

**Table 4:** Relative errors of evaluated residual stresses for plane shear stress state with α= 0° and 45°.

α (°)	$\sigma_{I}$ (MPa)	$\sigma_{\mathrm{II}}$ (MPa)	$\sigma_{I}/R_{e}$ (-)	${\bar{\sigma }}_{\mathrm{eq}}/R_{e}$ (-)	δ (%)	$\delta_{cor}$ (%)
0	144.3	–144.3	0.289	0.5	0.85	0.85
	173.2	–173.2	0.346	0.6	1.2	1.1
	230.9	–230.9	0.462	0.8	5.1	1.5
	259.8	–259.8	0.520	0.9	13.0	2.9
	274.2	–274.2	0.548	0.95	19.5	4.5
	288.7	–288.7	0.577	1	24.9	5.1
45	144.3	–144.3	0.289	0.5	0.85	0.85
	173.2	–173.2	0.346	0.6	1.2	1.1
	230.9	–230.9	0.462	0.8	5.2	1.6
	259.8	–259.8	0.520	0.9	13.1	3.0
	274.2	–274.2	0.548	0.95	19.5	4.6
	288.7	–288.7	0.577	1	24.4	4.9

**Table 5:** Relative errors of evaluated residual stress for plane shear stress state with α = 0°, 15°, 30°, 45° and 60° and the ratio ${\bar{\sigma }}_{eq}/R_{e}=0.8$.

$\sigma_{I}$ (MPa)	$\sigma_{\mathrm{II}}$ (MPa)	$\sigma_{I}/R_{e}$ (-)	${\bar{\sigma }}_{\mathrm{eq}}/R_{e}$ (-)	α (°)	δ (%)	$\delta_{cor}$ (%)
230.9	–230.9	0.462	0.8	0	5.1	1.5
				15	4.93	1.49
				30	5.0	1.5
				45	5.19	1.59
				60	5.0	1.5

**Table 6:** Relative errors of evaluated residual stress for equi-biaxial stress state.

$\sigma_{I}$ (MPa)	$\sigma_{\mathrm{II}}$ (MPa)	${\bar{\sigma }}_{\mathrm{eq}}/R_{e}$ (-)	$E_{T}$ (MPa)	δ (%)	$\delta_{cor}$ (%)
250	250	0.5	2100	–0.54	–0.54
300	300	0.6		–0.37	–0.76
400	400	0.8		5.7	–2.4
450	450	0.9		13.3	–3.1
475	475	0.95		17.3	–3.3
500	500	1		26.0	–1.6
